# CT imaging characteristics analysis of bone erosion in rheumatoid arthritis and bioinformatics study of inflammation-related gene rG4s

**DOI:** 10.3389/fmed.2026.1769517

**Published:** 2026-02-12

**Authors:** Mengyuan Chen

**Affiliations:** School of Public Health, Columbia University, New York, NY, United States

**Keywords:** bioinformatics, bone erosion, computed tomography imaging features, rG4s, rheumatoid arthritis

## Abstract

**Objective:**

This work aimed to collect joint computed tomography (CT) imaging and peripheral blood transcriptome data from patients with rheumatoid arthritis (RA), and construct a deep learning model for the automatic and precise assessment of bone erosion (BE). It was to screen RA-related inflammation genes regulated by rG4s through bioinformatics methods, explore potential associations between BE imaging phenotypes and molecular regulatory features, and provide hypotheses and clues for investigating the post-transcriptional regulatory mechanisms of RA bone destruction.

**Methods:**

Clinical data, joint CT images, and peripheral blood RNA sequencing data were collected from the RA group (AG, 148 cases) and the healthy control group (BG, 49 cases) at Yancheng Third People’s Hospital. DESeq2 software was used for differential expression analysis of RNA-seq data. Combined with an inflammation core gene set integrated from multiple databases, RA-related inflammation-related Differentially Expressed Genes (irDEGs) were screened. The rG4detector tool was used to predict rG4s structures in target genes. The Metascape database was used for functional enrichment analysis to identify core candidate genes. An optimized U-Net CNN model was constructed based on the PyTorch framework to achieve automatic segmentation and severity quantification of BE in CT images. Multiple metrics were used to evaluate model performance, and the correlation between candidate gene expression levels and imaging scores was analyzed.

**Results:**

A total of 67 RA-related irDEGs were screened, of which 42 contained potential rG4s structures. The U-Net CNN model performed excellently in BE segmentation, with pixel-level accuracy, Dice Similarity Coefficient (DSC), sensitivity, and specificity on the test set all at high levels. The model’s quantitative score was significantly correlated with the clinical disease activity score (DAS28).

**Conclusion:**

CT imaging characteristics of BE in RA patients were closely associated with the expression of rG4s-regulated irDEGs. The deep learning model constructed in this study enabled precise quantification of BE, providing an efficient method for the clinical assessment of RA bone erosion. It also offered a new research perspective and candidate targets for understanding the molecular mechanisms of RA bone destruction at the post-transcriptional regulatory level.

## 1 Introduction

Rheumatoid arthritis (RA) is a common chronic autoimmune disease characterized by persistent inflammatory response and progressive joint structural destruction. Bone erosion (BE), as a core pathological change in disease progression, can occur early and gradually worsen, directly leading to joint deformity, functional impairment, and even disability (1, 2), severely affecting patients’ quality of life. Timely and accurate assessment of the presence and severity of BE is of great significance for disease staging, treatment adjustment, and prognosis evaluation (3).

Computed tomography (CT), with its advantages of high spatial resolution and excellent bone tissue display, can clearly show the location, extent, and morphological features of BE, and has become an important imaging method for clinically assessing BE in RA patients (4). However, traditional assessment methods rely on physicians’ subjective judgment, which is time-consuming, inefficient, and influenced by individual experience differences, leading to poor inter-observer consistency (5, 6) and inconsistent evaluation results across different physicians and medical institutions. This subjectivity and inefficiency limit its widespread application in large-scale clinical research and routine diagnosis and treatment, highlighting the urgent need to develop more objective and efficient automated assessment methods.

The occurrence of BE is a complex pathological process related to inflammatory response, immune cell activation, osteoclast dysfunction, and other aspects (7). Its underlying molecular mechanisms are not yet fully understood. Some genes are directly involved in regulating bone metabolic balance, affecting osteoclast differentiation, maturation, and bone resorption function, thereby promoting the occurrence and development of BE (8–10). RNA G-quadruplexes (rG4s) are special secondary structures formed by folding of guanine-rich RNA sequences, widely existing in different regions of genes. They can affect gene function through various means such as post-transcriptional regulation and translation processes (11, 12) and may play an important role in the pathological processes of inflammatory and immune-related diseases (13). However, current research on rG4s in RA-related BE is relatively scarce, and the mechanisms by which they influence BE occurrence and development remain unclear. Whether there is an association between BE imaging features and the expression of rG4s-regulated inflammation-related genes has not been systematically explored.

Based on the current research status, this study integrated deep learning technology and bioinformatics methods to systematically analyze the CT imaging characteristics of BE in RA patients and inflammation-related gene rG4s. A deep learning model was used to achieve automatic recognition and quantitative assessment of BE in CT images. Bioinformatics techniques were used to screen RA-related inflammation-related differentially expressed genes (irDEGs), and their rG4s characteristics and functional enrichment were analyzed. The study aimed to establish associations between imaging phenotypes and molecular regulatory features, providing a more precise and efficient assessment tool for BE in RA patients.

## 2 Materials and methods

### 2.1 Study subjects

This study was a retrospective analysis. Data were sourced from the medical system of Yancheng Third People’s Hospital. Clinical data, joint CT images, and peripheral blood RNA sequencing data from RA patients and healthy individuals undergoing physical examinations between January 2020 and December 2023 were collected. All procedures in this study were approved by the Ethics Committee of Yancheng Third People’s Hospital and complied with international ethical guidelines such as the Declaration of Helsinki. As this was a retrospective analysis, all data had been de-identified. Informed consent was waived by the Ethics Committee, so participants were not required to sign consent forms.

*Inclusion criteria for the RA group (AG)*: Met the 2010 American College of Rheumatology (ACR)/European Alliance of Associations for Rheumatology (EULAR) classification criteria for RA (14); aged 18–75 years, regardless of gender; complete clinical data including medical history, laboratory tests, and treatment records; completed joint CT scans of commonly affected sites [wrist, metacarpophalangeal joints (MPJ), proximal interphalangeal joints (PIPJ)] at Yancheng Third People’s Hospital; peripheral blood samples were collected and RNA sequencing was completed. A total of 148 RA patients were included, with 103 assigned to the CT image training set, 29 to the validation set, and 16 to the test set. All patients underwent qualified RNA sequencing.

*Inclusion criteria for the healthy control group (BG)*: Matched 1:1 with AG in terms of age and gender; no history of rheumatic immune diseases, no symptoms such as joint pain or swelling; negative laboratory results for rheumatoid factor and anti-cyclic citrullinated peptide antibody; completed CT scans of the same joint sites, showing normal joint structure without BE or osteophyte formation; peripheral blood samples were collected and RNA sequencing was completed. A total of 49 healthy controls were included, with 34 assigned to the CT image training set, 10 to the validation set, and 5 to the test set. RNA sequencing data for all subjects were qualified.

*Exclusion criteria*: Comorbidities with other rheumatic immune diseases affecting joint structure; presence of severe hepatic/renal insufficiency, malignancy, or coagulation dysfunction; joint surgery, glucocorticoid pulse therapy, or adjustment of immunosuppressants within 6 months before CT scan; incomplete or blurred imaging data; RNA sample degradation (RNA Integrity Number < 7.0) or substandard sequencing data (sequencing depth < 6G or alignment rate < 85%); pregnancy or lactation.

### 2.2 Data collection content and specifications

Clinical data collection covered general information of all participants (age, sex, height, weight, and disease duration). Relevant laboratory test indicators were also obtained, including rheumatoid factor titer, anti-cyclic citrullinated peptide antibody levels, C-reactive protein, erythrocyte sedimentation rate, and the clinical disease activity score (DAS28).

CT imaging data were acquired from the hospital’s GE Discovery CT750 HD 64-slice spiral CT scanner. Scan parameters: tube voltage 120 kV, tube current 200-300 mA, slice thickness 0.625 mm, slice spacing 0.625 mm, matrix 512 × 512, field of view 12-15 cm. The scan range fully covered both wrists, all MPJ, and PIPJ. The data were stored in DICOM format.

RNA sequencing data were derived from 5 mL of peripheral venous blood collected and preserved in EDTA anticoagulant tubes. Extraction of total RNA was carried out adopting Trizol reagent, with purity tested by Nanodrop 2000, requiring an A260/A280 ratio between 1.8 and 2.0. RNA intactness was verified via the Agilent 2100 Bioanalyzer, with values not lower than 7.0. Sequencing was implemented on the Illumina NovaSeq 6000 platform in paired-end mode with a read length of 150 bp, and the sequencing depth for each sample was required to be above 6G. The raw sequencing data were saved in FASTQ format.

### 2.3 Data preprocessing

Regarding CT image preprocessing, processing DICOM files was carried out via Python 3.8 and the Simple ITK library. Conversion of DICOM files to the NIfTI format suitable for deep learning was executed. Subsequently, to enhance image contrast, adaptive histogram equalization was applied, which yielded clearer boundaries between bone and soft tissues. Determination of CT value ranges was carried out for preliminarily extracting bone tissue regions and removing non-target tissues (skin, muscle, etc.). The images were subjected to rigid registration into a standard anatomical coordinate system for comparability of joint positions across all participants. Finally, resampling all images to isotropic voxels of uniform size was executed to minimize the impact of scale differences on subsequent analyses. The final image data were saved in NIfTI format.

For RNA sequencing data preprocessing, Fast QC software was used to assess the quality of the raw sequencing data and filter out substandard sequences, including those with adapter sequences, high proportions of unknown bases, and high proportions of low-quality bases. Subsequently, HISAT2 software was employed to align these high-quality reads to the human reference genome, retaining samples with acceptable alignment rates. Finally, Feature Counts software was used to calculate the read counts for each gene, generating a gene expression matrix that would be used for the subsequent differential expression analysis.

### 2.4 Differential expression of inflammatory genes and rG4 structure prediction

#### 2.4.1 Construction of inflammation-related gene set

Based on Gene Cards, OMIM, and Reactome pathway databases, RA-related inflammatory genes were retrieved and integrated using the keywords “RA,” “inflammation,” “BE,” and “immune response.” Duplicate genes were removed, and genes supported by at least three publications were selected, ultimately resulting in a core set of 826 inflammation-related genes.

#### 2.4.2 Differentially expressed genes (DEGs) screening

The preprocessed RNA-seq gene expression matrix (R language and DESeq2 package) was applied. The gene read count matrix was imported into DESeq2 to construct a design model with “sample type” (AG/BG) as the variable. Differential expression testing was then conducted using the DESeq function, with screening thresholds set at |log_2_FC| ≥ 1.5 and false discovery rate (FDR) < 0.01. Subsequently, hierarchical clustering analysis was performed on the DEGs using Euclidean distance and Ward’s method, and the differential expression features were visualized using volcano plots and heatmaps. Finally, an intersection analysis was conducted between the differential expression results and the previously constructed inflammation-related core gene set to identify RA-associated irDEGs.

#### 2.4.3 rG4 structure prediction and feature analysis

The rG4detector tool was used to predict rG4 structures in the mRNA sequences of irDEGs. Parameter settings were as follows: complete mRNA sequences of target genes, including 5’UTR, coding region (CDS), and 3’UTR, were obtained from the Ensembl database; the rG4 core sequence motif was set to G ≥ 3, loop length 1–7 nt, prediction window size 20 nt; default threshold (probability ≥0.5) was used to screen genes containing potential rG4 structures. Sensitivity analysis was performed by adjusting the threshold (0.4–0.6), showing no significant change in the core rG4-positive gene set, indicating stable prediction results (Table 1). The annotation module of rG4detector was used to count the number of rG4s per gene, their locations (5’UTR/CDS/3’UTR), number of core G-quadruplexes, and loop length distribution.

**Table 1:** Sensitivity analysis results of positive gene sets under different rG4 prediction thresholds.

Threshold (probability)	Total irDEGs number	rG4s positive gene number	Positive rate (%)	Intersection number with default threshold (0.5) positive genes	Intersection rate (%)	Core positive gene Set (top15) retained number	Core gene retention rate (%)
0.4 (Lenient threshold)	67	46	68.66	42	95.45	15	100
0.5 (Default threshold)	67	42	62.69	42	100	15	100
0.6 (Strict threshold)	67	38	56.72	38	90.48	14	93.33

#### 2.4.4 Functional enrichment analysis

GO functional (biological process, BP; cellular component, CC; molecular function, MF) and KEGG pathway enrichment (centered on pathways correlated with immune regulation, inflammatory response, and bone metabolism) analyses via the Metascape database elucidated the biological functions of irDEGs having rG4 structures. The screening threshold was FDR < 0.05, with at least three genes per enriched term. The interaction network of enriched pathways was visualized via Cytoscape software (version 3.10.2), node size represented the number of genes, and edge weight represented the degree of gene overlap between pathways. Core functional pathways were identified accordingly.

### 2.5 Image annotation and dataset division

#### 2.5.1 BE annotation standards

BE annotation standards: pathological BE: presence of cortical bone disruption in at least two consecutive slices in the vertical plane, along with the loss of underlying trabecular bone and a non-linear shape. Physiological cortical bone defects and vascular channels were excluded. The annotation content included pixel-level segmentation masks of BE areas, number, maximum diameter (measured in both the vertical and axial planes), and the location of the affected joint (wrist, MPJ, or PIPJ).

#### 2.5.2 Annotation process

Two physicians with over 5 years of experience in rheumatologic and immunologic imaging diagnosis served as annotators. They used 3D Slicer software to blindly annotate the preprocessed CT images. Before annotation, a training session was conducted using 10 reserved images for pre-annotation calibration. Each annotator independently completed the segmentation of BE areas and recorded the parameters for all images. Intraclass correlation coefficient (ICC) was used to assess annotation consistency. For regions with ICC < 0.75, a third senior physician with over 10 years of experience made the final arbitration to form the gold standard annotation set. The final ICC value for the annotations in this study was 0.82 (95% CI: 0.76–0.87), which met the requirements.

#### 2.5.3 Dataset division

The preprocessed and annotated CT image data were randomly allocated to training set (137 cases, AG: 103, BG: 34), validation set (39 cases, AG: 29, BG: 10), and test set (21 cases, AG: 16, BG: 5).

### 2.6 Construction of deep learning CNN model

#### 2.6.1 U-Net CNN

Based on the PyTorch framework, an optimized U-Net CNN model for medical image segmentation was constructed. The core structure was an encoder-decoder architecture capable of precise localization and segmentation of BE regions. Model innovations were as follows:

- Contraction path (feature extraction module): Consisted of 4 down sampling CNN units. Each unit contained 2 unpadded 3 × 3 convolutional layers (Conv2d). The convolution operation was defined as: $y_{i,j}=\sigma (\sum_{k=1}^{C_{\text{in}}}\sum_{p=0}^{2}\sum_{q=0}^{2}\omega_{k,p,q}\cdot x_{k,i+p,j+q}+b)$ . $y_{i,j}$ represents the output feature image pixel value, σ is the ReLU activation function, $C_{\text{in}}$ is the number of input channels, $\omega_{k,p,q}$ is the convolution kernel weight, $x_{k,i+p,j+q}$ is the input feature image pixel value, and b is the bias term. Each CNN unit was followed by a 2 × 2 max pooling layer (stride = 2) for down sampling. The channel count increased sequentially from 64 to 512. Dropout layers (dropout rate 0.5) were added after the 3rd and 4th units to reduce overfitting by randomly deactivating neurons.
- Expansion path (feature reconstruction module): Consisted of 4 upsampling CNN units. Each unit first enlarged the feature map size via a 2 × 2 transposed convolutional layer (ConvTranspose2d). The transposed convolution operation was defined as: $y_{i,j}=\sigma (\sum_{k=1}^{C_{\text{in}}}\sum_{p=0}^{2}\sum_{q=0}^{2}\omega_{k,p,q}\cdot x_{k,i-p,j-q}+b)$ _._ Subsequently, this feature map was concatenated with the corresponding feature map from the contraction path to compensate for boundary features lost during down sampling, followed by further feature optimization through two 3 × 3 convolutional layers.
- Output layer: A 1 × 1 convolutional layer was used to map the 512-channel feature map into two classes: background and BE region. The Softmax function was applied to output pixel-level classification probabilities, defined as: $P(y=k|x)=\frac{e^{z_{k}}}{\sum_{m=1}^{2}e^{z_{m}}}$ . $z_{k}$ . Represents the logits value output by the 1 × 1 convolutional layer, and P(y=k∣x) represents the likelihood of a pixel pertaining to class *k*.

#### 2.6.2 Data augmentation strategy

The data augmentation strategy was employed to enhance the generalization capability of the convolutional neural network model. Online augmentation was adopted to images in the training set. Geometric transformations included random rotation within −15 to 15 degrees, translation by ±10 pixels, scaling from 0.8 to 1.2 times, and horizontal flipping. Intensity augmentation involved randomly adjusting contrast within 0.8 to 1.2 times, brightness changes by ±10 HU, and adding Gaussian noise with a variance of 0.01. Elastic deformation was applied by imposing random displacement vectors on a 3 × 3 grid, with the vectors following a Gaussian distribution and a standard deviation of 10 pixels. Bicubic interpolation was used to achieve smooth deformation.

#### 2.6.3 Training parameter settings

The AdamW optimizer was adopted (initial learning rate of 1e-4). A cosine annealing learning rate schedule was applied (*T*_max_ = 50, eta_min_ = 1e-6), and the weight decay coefficient was 1e-5.

A weighted sum of Dice loss and cross-entropy loss was used, with a weight ratio of 1:1. $\text{Dice}=1-\frac{2\cdot |X\cap Y|+\in }{|X|+|Y|+\in }$ . X . Represents the model-predicted segmentation area, and Y represents the gold standard annotated area, with e=1e−6 added to avoid division by zero.

During training, a batch size of 4 and 100 epochs were adopted. Early stopping was implemented with a patience of 10 epochs based on the validation loss, and the optimal model weights were saved. NVIDIA A100 GPUs were used for model training and inference acceleration.

### 2.7 Model performance evaluation

#### 2.7.1 Segmentation performance metrics

The segmentation performance of the CNN model for BE areas was evaluated on the test set using the following four metrics:

- Pixel-level Accuracy: Proportion of correct classifications out of the total count.
- Dice Similarity Coefficient (DSC): A measure of the overlap between the predicted area and the gold standard, with values approaching 1 denoting superior segmentation performance.
- Sensitivity: Proportion of correctly identified BE pixels in total BE pixels in the gold standard, which reflects the identification capability.
- Specificity: Proportion of correctly identified background pixels in total background pixels in the gold standard, which reflects the model’s ability to distinguish normal tissues.

#### 2.7.2 Severity scoring and grouping

Based on the segmentation results of the CNN model, a BE severity assessment system was established. The weight allocation for this system was based on: referencing previous bone erosion scoring criteria (6), integrating opinions from three clinical rheumatology specialists, and determining indicator weights using the Analytic Hierarchy Process (AHP); after preliminary validation (involving CT image data from 20 RA patients, comparing scoring discriminative power under different weight combinations), the final core evaluation indicators were determined as: number of lesions (weight 0.3), average maximum diameter of all lesions (weight 0.4), and ratio of erosion region volume to total joint bone tissue volume (weight 0.3). A quantitative score (0–100) reflecting the overall severity of BE was calculated via linear weighting.

In addition to the overall score, the system also included local severity scores for specific joints (wrist, MPJ, PIPJ, etc.), also generating quantitative results (0–100), used to assess the local erosion status of each joint.

Based on the overall score, patients were divided into no BE, mild BE, moderate BE, and severe BE groups. By comparing the rate of score change in multiple follow-up images and abnormally high local scores, rapid progression groups and special site erosion groups were defined.

#### 2.7.3 Clinical consistency validation

The intraclass correlation coefficient (ICC) was used to evaluate the consistency between the CNN model predictions and the gold standard annotations by two senior physicians. The Pearson correlation coefficient (PCC) was used to verify the clinical correlation between model scores and DAS28. Normality of data for correlation analysis was confirmed by the Shapiro–Wilk test, showing all data conformed to a normal distribution, meeting the assumptions for Pearson correlation analysis. Statistical analysis was performed using SPSS 27.0 software, with a significance level of *α* = 0.05.

## 3 Results

### 3.1 Construction results of the inflammation-related core gene set

After integrating and screening through multiple databases, a final set of 826 genes was established as the RA-related inflammation core gene set. This gene set encompasses key genes related to inflammation regulation, immune response, and bone metabolism in the pathological process of RA, providing a reliable pool of target genes for subsequent differential expression analysis (Table 2).

**Table 2:** Representative genes in the inflammation-related core gene set (Top 20).

Gene symbol	Gene name	UniProt ID
TNF	Tumor necrosis factor	P01375
IL6	Interleukin 6	P05231
IL1β	Interleukin 1 beta	P01584
CCL2	C-C motif chemokine ligand 2	P13501
MMP9	Matrix metalloproteinase 9	P14780
RANKL	Receptor activator of nuclear factor kappa-b ligand	O14788
IFNG	Interferon gamma	P01579
IL17A	Interleukin 17A	Q16552
OPG	Osteoprotegerin	O00300
CXCL8	C-X-C motif chemokine ligand 8	P10145
IL10	Interleukin 10	P22301
TGFB1	Transforming growth factor beta 1	P01137
IL18	Interleukin 18	Q14116
CCR5	C-C Chemokine receptor type 5	P51681
MMP3	Matrix metalloproteinase 3	P08254
IL4	Interleukin 4	P05112
IL2	Interleukin 2	P60568
STAT3	Signal transducer and activator of transcription 3	P40763
NFKB1	Nuclear factor kappa-B subunit 1	P19838
VEGFA	Vascular endothelial growth factor A	P15692

### 3.2 Results of differential expression analysis of RA-related inflammatory genes

**Figure 1:**
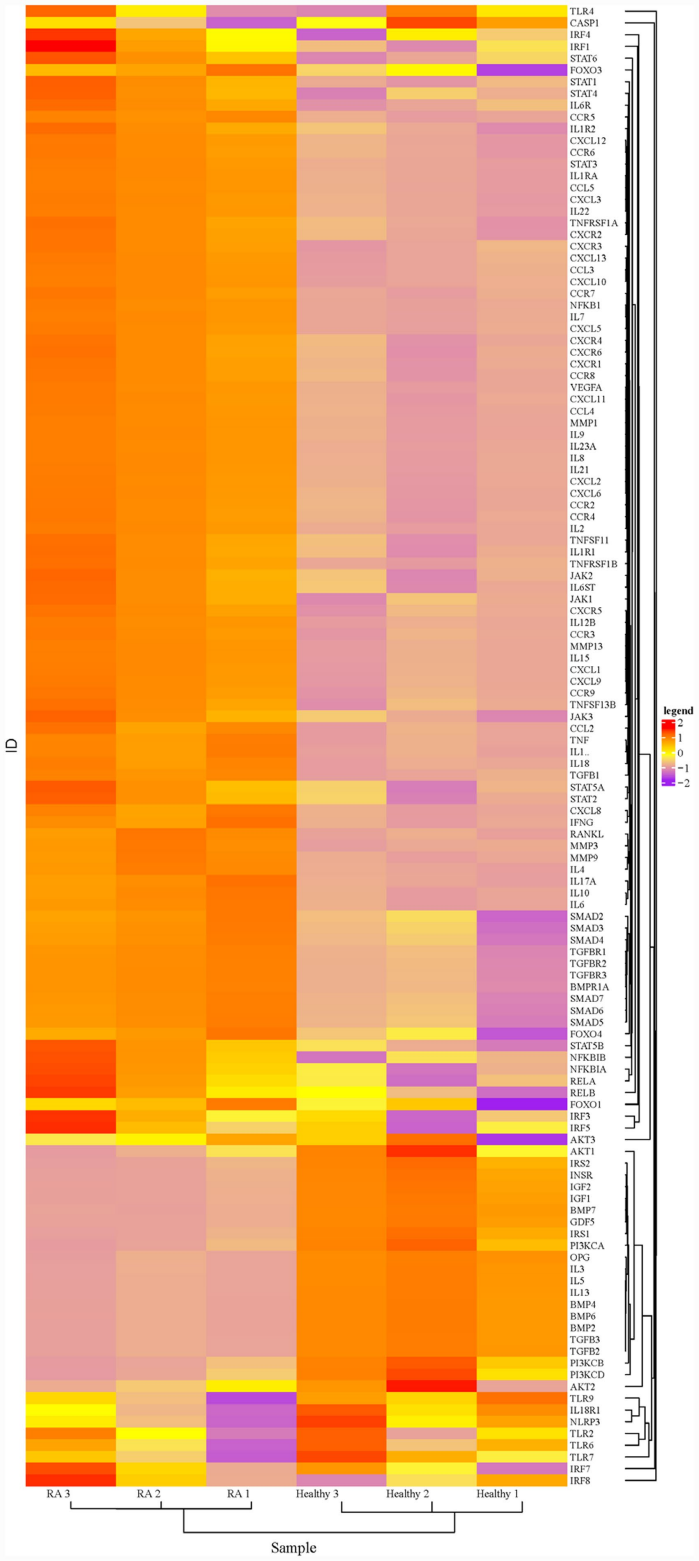
Hierarchical clustering heatmap of DEGs. The *x*-axis represents samples, and the *y*-axis represents DEGs; the color gradient indicates the normalized values of gene expression levels (red, high expression, purple, low expression).

**Figure 2:**
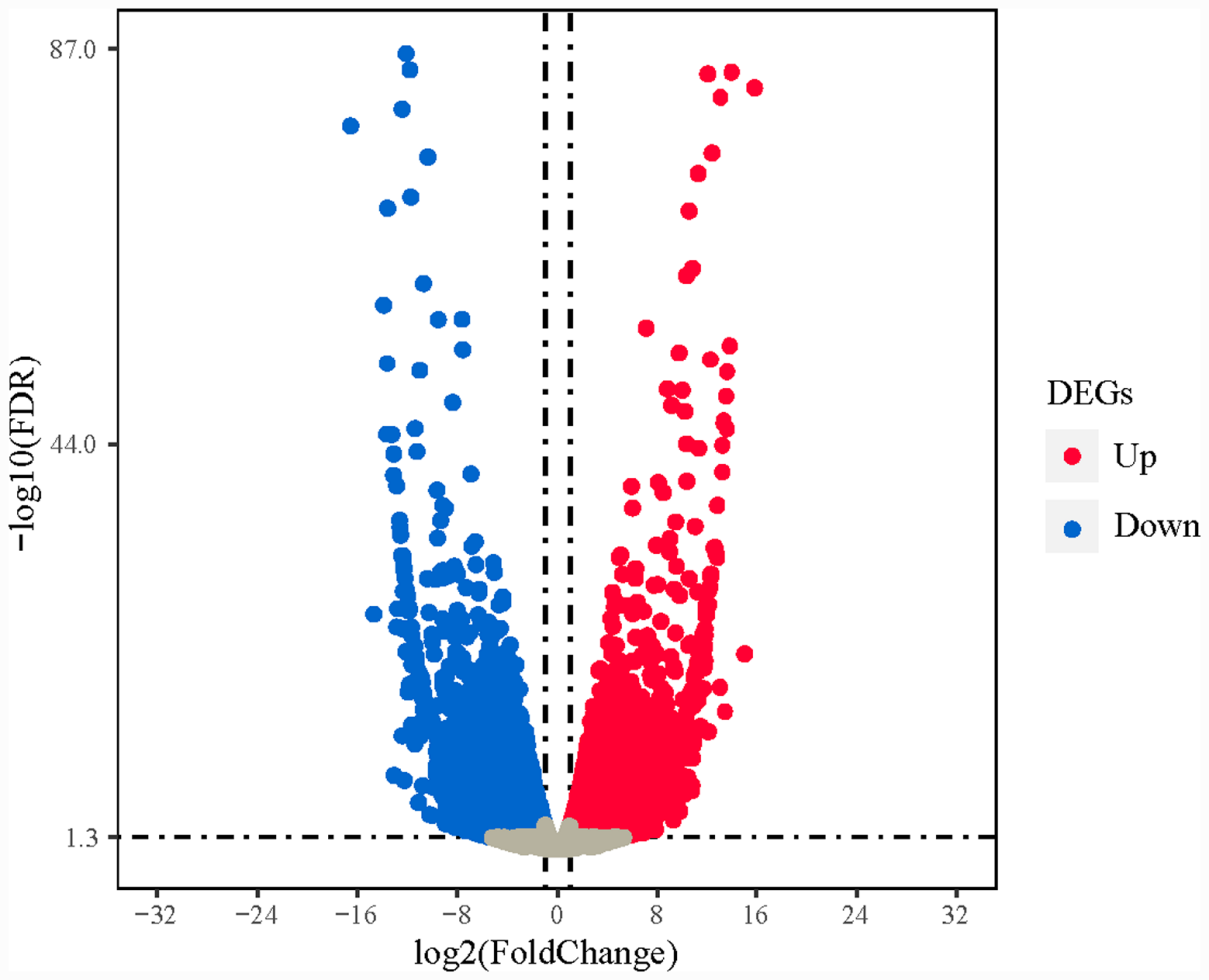
Volcano plot of DEGs. *x*-axis, log₂FC value; *y*-axis, -log₁₀(FDR); red nodes, upregulated DEGs; blue nodes, downregulated DEGs; gray nodes, genes with no significant difference.

A total of 128 differentially expressed genes (DEGs) were screened, with 93 upregulated and 35 downregulated. Hierarchical clustering analysis (Figure 1) showed significant differences in DEG expression clustering patterns between AG and BG, clearly distinguishing the two sample groups. A volcano plot displayed the differential expression distribution characteristics of all genes, with red nodes representing significantly upregulated DEGs, purple nodes representing significantly downregulated DEGs, and yellow nodes representing genes with no significant difference (Figure 2).

The 128 DEGs were intersected with the inflammation-related core gene set constructed in section 3.1, ultimately yielding 67 RA-related inflammation-related DEGs (irDEGs), with 52 upregulated and 15 downregulated. These genes are centrally involved in the inflammatory response and bone metabolism regulation processes of RA, providing a core target gene set for subsequent rG4 structure prediction.

### 3.3 Results of rG4s structure prediction and feature analysis of irDEGs

The mRNA sequences of the 67 irDEGs involving RA were subjected to rG4s structure prediction using the rG4detector tool. With a prediction probability of ≥0.5 as the criterion, a total of 42 rG4s-positive genes containing potential rG4s structures were identified among the 67 irDEGs, with a positivity rate of 62.69%. Among the 52 upregulated irDEGs, 31 were rG4s-positive, with a positivity rate of 59.62%; among the 15 downregulated irDEGs, 11 were rG4s-positive, with a positivity rate of 73.33%. The distribution density of rG4s structures in downregulated genes was slightly higher than that in upregulated genes (Table 3).

**Table 3:** Number and location distribution of rG4s in representative rG4s-positive genes.

Gene symbol	Differential expression trend	Number of rG4s	Distribution of rG4s locations
TNF	Upregulated	2	CDS, 3’UTR
IL6	Upregulated	3	5’UTR, CDS, 3’UTR
IL1β	Upregulated	1	3’UTR
MMP9	Upregulated	5	5’UTR (2), CDS (1), 3’UTR (2)
RANKL	Upregulated	2	CDS, 3’UTR
OPG	Downregulated	3	5’UTR, 3’UTR (2)
TGFB1	Upregulated	1	CDS
CCL2	Upregulated	2	3’UTR (2)
IL17A	Upregulated	1	5’UTR
SMAD3	Downregulated	2	CDS, 3’UTR
IFNG	Upregulated	2	CDS, 3’UTR
CXCL8	Upregulated	1	3’UTR
STAT3	Upregulated	3	CDS (2), 3’UTR
NFKB1	Upregulated	2	5’UTR, CDS
BMP2	Downregulated	1	3’UTR

The locations of rG4s in the 42 rG4s-positive genes were annotated and analyzed, revealing that they were distributed across the 5’UTR, CDS, and 3’UTR of the mRNA, with significant differences in distribution frequency among different regions. The number of rG4s per gene exhibited diverse characteristics, with some genes having multiple rG4s structures in multiple regions (Tables 4, 5).

**Table 4:** Distribution statistics of rG4s structures in different regions of mRNA.

mRNA region	Number of rG4s elements	Percentage in total rG4s elements (%)
5’UTR	23	17.97
CDS	47	36.72
3’UTR	58	45.31
Total	128	100

**Table 5:** Statistics on the distribution of rG4s in single genes.

Number of rG4s	Number of genes	Proportion in positive genes (%)
1	19	45.24
2	14	33.33
≥3	9	21.43
Total	42	100

It must be emphasized that all rG4 structure prediction results are bioinformatics computational predictions. Their actual functions require subsequent experimental validation.

The core structural parameters of the 128 rG4s elements in the 42 positive genes were statistically analyzed. Both the distribution of the number of core G-quadruplexes and the loop length showed a distinct clustering tendency (Tables 6, 7). The results indicated that rG4s structures were commonly found in the irDEGs associated with RA, and were mainly located in the 3’UTR and CDSs. The core structure was characterized by three G-quadruplexes and 3-5 nt of loop length. These features regulate gene post-transcriptional expression or translation.

**Table 6:** Statistics on the distribution of core G-quadruplex numbers in rG4s structures.

Number of core G-quadruplexes	Number of rG4s elements	Proportion in total rG4s elements (%)
2	7	5.47
3	89	69.53
4	32	25
≥5	0	0
Total	128	100

**Table 7:** Statistics on the distribution of loop lengths in rG4s structures.

Loop length (nt)	Number of rG4s elements	Proportion in total rG4s elements (%)
1	8	6.25
2	13	10.16
3	41	32.03
4	35	27.34
5	24	18.75
6	5	3.91
7	2	1.56
Total	128	100

### 3.4 Functional enrichment analysis results of irDEGs containing rG4s structures

The GO and KEGG enrichment analyses were conducted on the 42 irDEGs involving RA containing rG4s structures, and a pathway interaction network was drawn to clarify the functional positioning and pathway association characteristics of the genes. The GO functional enrichment analysis screened out significantly enriched items in the three dimensions, with the core functions focusing on the key links in the RA pathological process.

**Figure 3:**
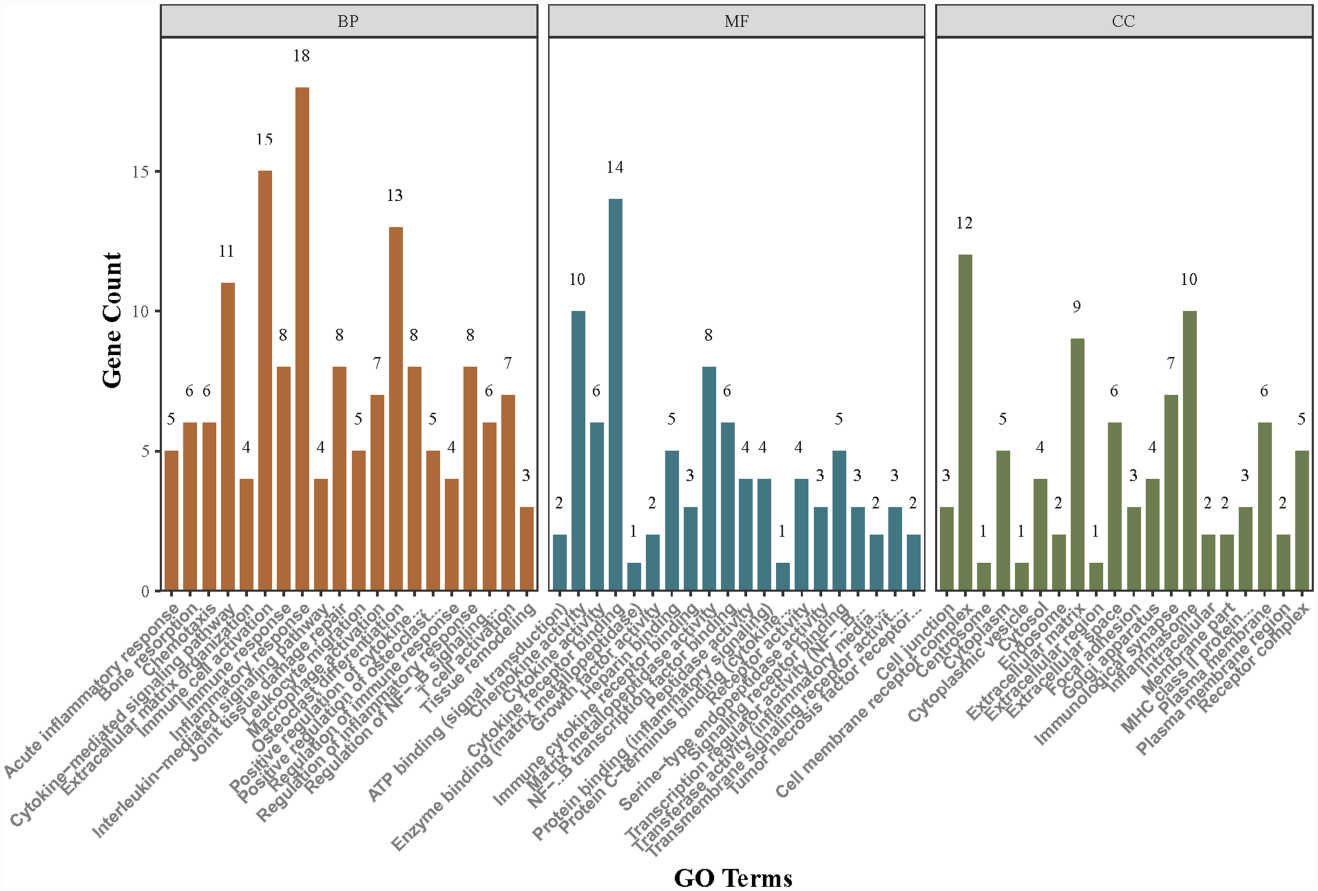
GO functional enrichment.

Figure 3 shows the top 20 enriched items of GO functions. The core items in the BP dimension were mainly enriched in inflammatory response (GO:0006954, 18 enriched genes), immune cell activation (GO:0002227, 15 enriched genes), osteoclast differentiation (GO:0030316, 11 enriched genes), cytokine-mediated pathway (GO:0019221, 13 enriched genes), and joint tissue damage repair (GO:0042060, 8 enriched genes), indicating that the genes containing rG4s structures were centrally involved in the RA inflammatory regulation and bone metabolism disorder process. The CC dimension was significantly enriched in cell membrane receptor complex (GO:0009986, 12 enriched genes), inflammasome (GO:0061630, 9 enriched genes), extracellular matrix (GO:0031012, 10 enriched genes), and immune synapse (GO:0001772, 7 enriched genes), reflecting that the functional positioning of these genes was closely correlated with inflammatory signal transduction and intercellular interaction. The MF dimension was mainly enriched in cytokine receptor binding (GO:0005126, 14 enriched genes), chemokine activity (GO:0008009, 10 enriched genes), matrix metalloproteinase activity (GO:0004222, 8 enriched genes), and NF-κB transcription factor binding (GO:0044183, 6 enriched genes), demonstrating their key roles in inflammatory molecular recognition, signal transduction, and tissue remodeling.

**Figure 4:**
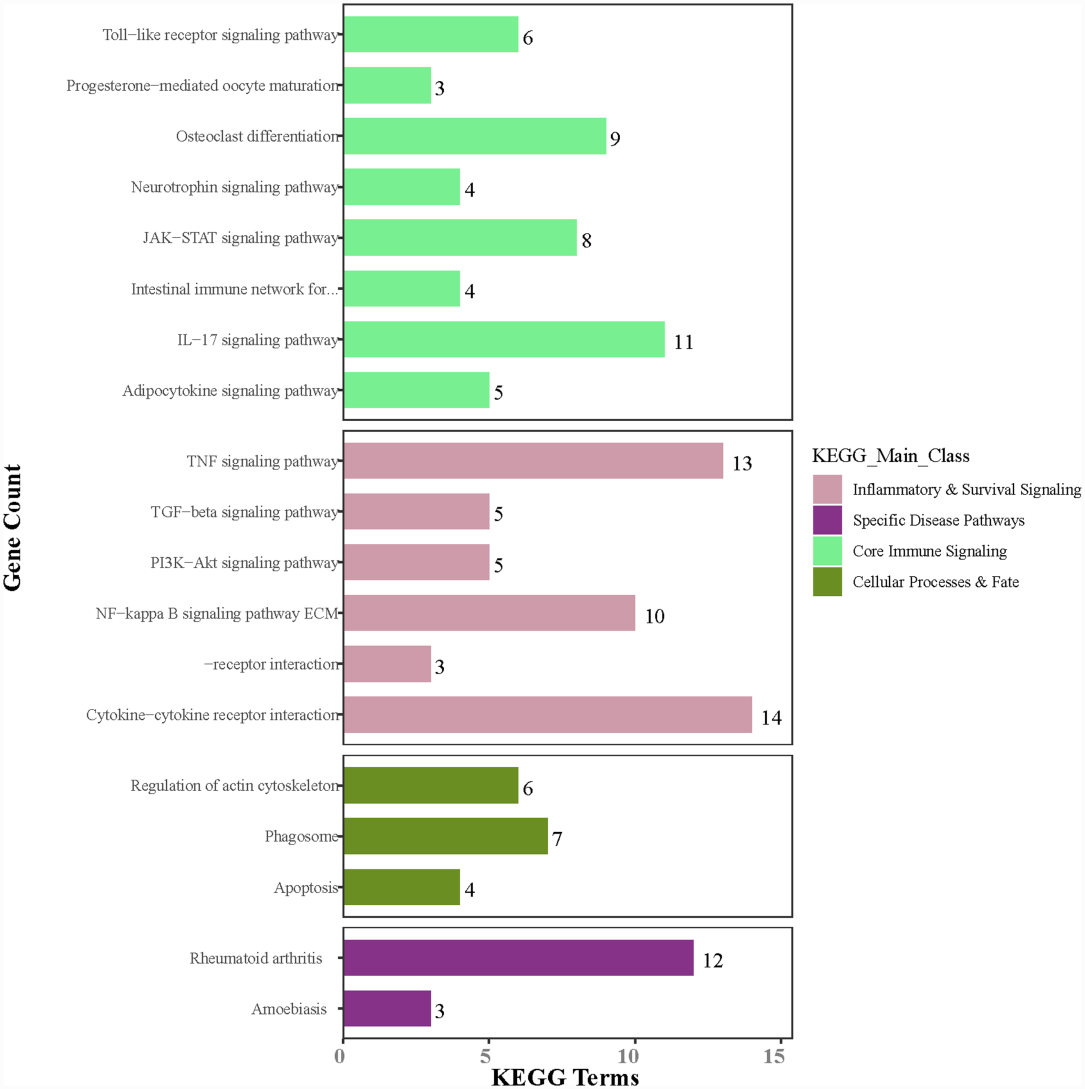
KEGG pathway enrichment.

A total of 19 noticeably enriched pathways were obtained, closely related to RA inflammatory regulation and bone metabolism. The TNF pathway had the highest number of enriched genes (13) and the smallest FDR value (1.8 × 10^−12^) (Figure 4).

**Figure 5:**
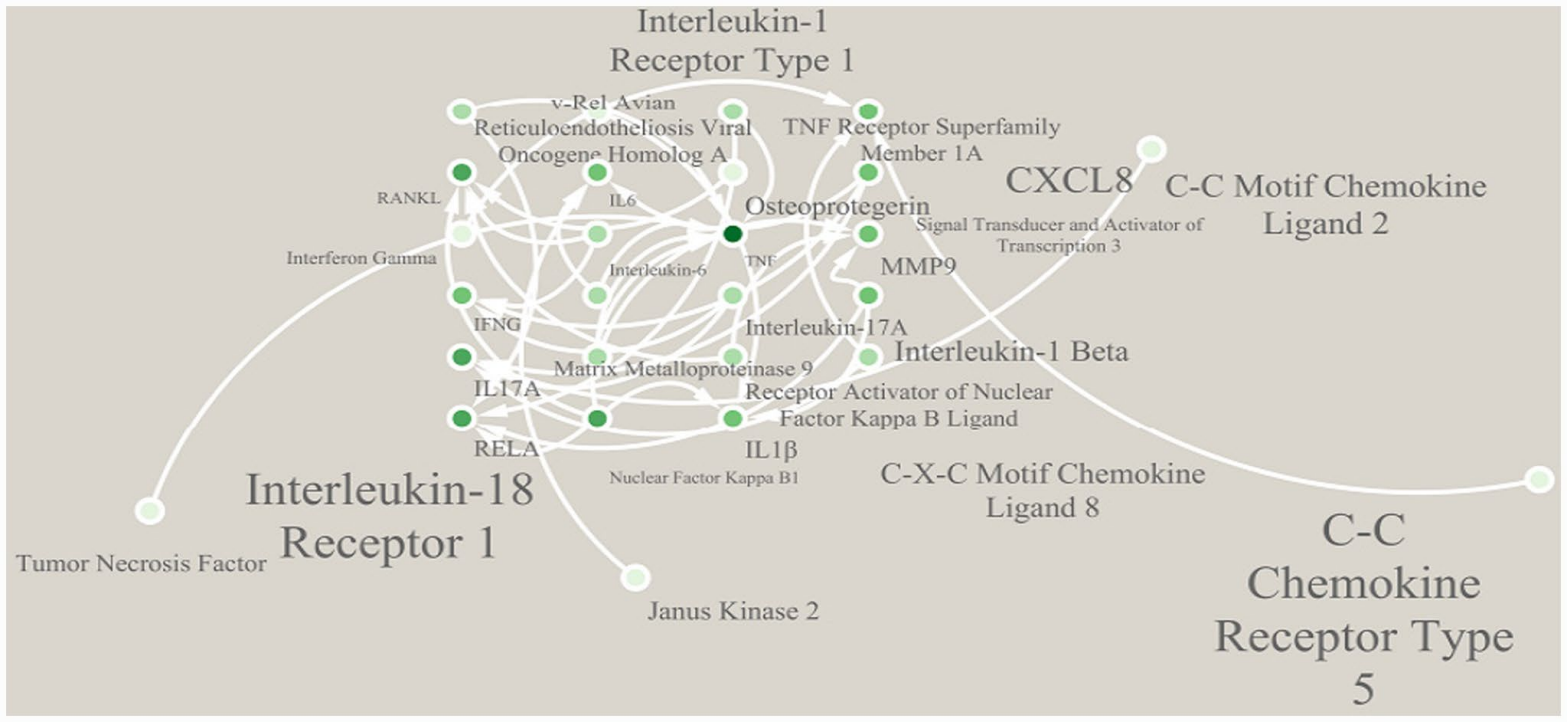
Interaction network of enriched pathways.

The pathway interaction network diagram displayed the relationships between core pathways and the network hubs. The core interaction module included RA pathway, IL-17 pathway, etc. The TNF pathway served as the network hub node, having direct connections with seven pathways. The genes enriched in this pathway, such as TNF, IL6, and MMP9, were also involved in the regulation of multiple pathways, mediating the cascade reactions of inflammation amplification and BE (Figure 5). Similarly, the regulatory effects of rG4s on these pathways are bioinformatics predictions requiring experimental validation.

### 3.5 Evaluation results of BE segmentation capability of the model

Based on the 21 CT imaging data in the test set, four indicators were adopted for evaluation of the BE segmentation performance of the model. The model had good segmentation and lesion detection abilities (Table 8).

**Table 8:** Model segmentation performance evaluation results (test set).

Evaluation indicator	Value	95% CI
Pixel-level accuracy	98.7 ± 0.6%	98.2–99.2%
DSC	0.863 ± 0.042	0.841–0.885
Sensitivity	84.7% ± 5.1%	82.0–87.4%
Specificity	99.1% ± 0.4%	98.9–99.3%

The segmentation performance of the model was analyzed by stratifying according to the joint locations. The results showed that the model achieved stable and reliable segmentation effects in wrist joint, MPJ, and PIPJ. The highest DSC value was obtained in the wrist joint. Due to the relatively complex anatomical structure, the sensitivity in the PIPJ was slightly lower than that in other locations, but it still remained above 80% (Table 9).

**Table 9:** Model segmentation performance indicators for different joint locations (test set).

Joint location	Pixel-level accuracy	DSC	Sensitivity	Specificity
Wrist joint	98.9% ± 0.5%	0.881 ± 0.036	86.5% ± 4.7%	99.2% ± 0.3%
MPJ	98.6% ± 0.7%	0.859 ± 0.045	84.3% ± 5.3%	99.0% ± 0.5%
PIPJ	98.4% ± 0.8%	0.838 ± 0.048	81.6% ± 5.8%	98.9 ± 0.6%

**Figure 6:**
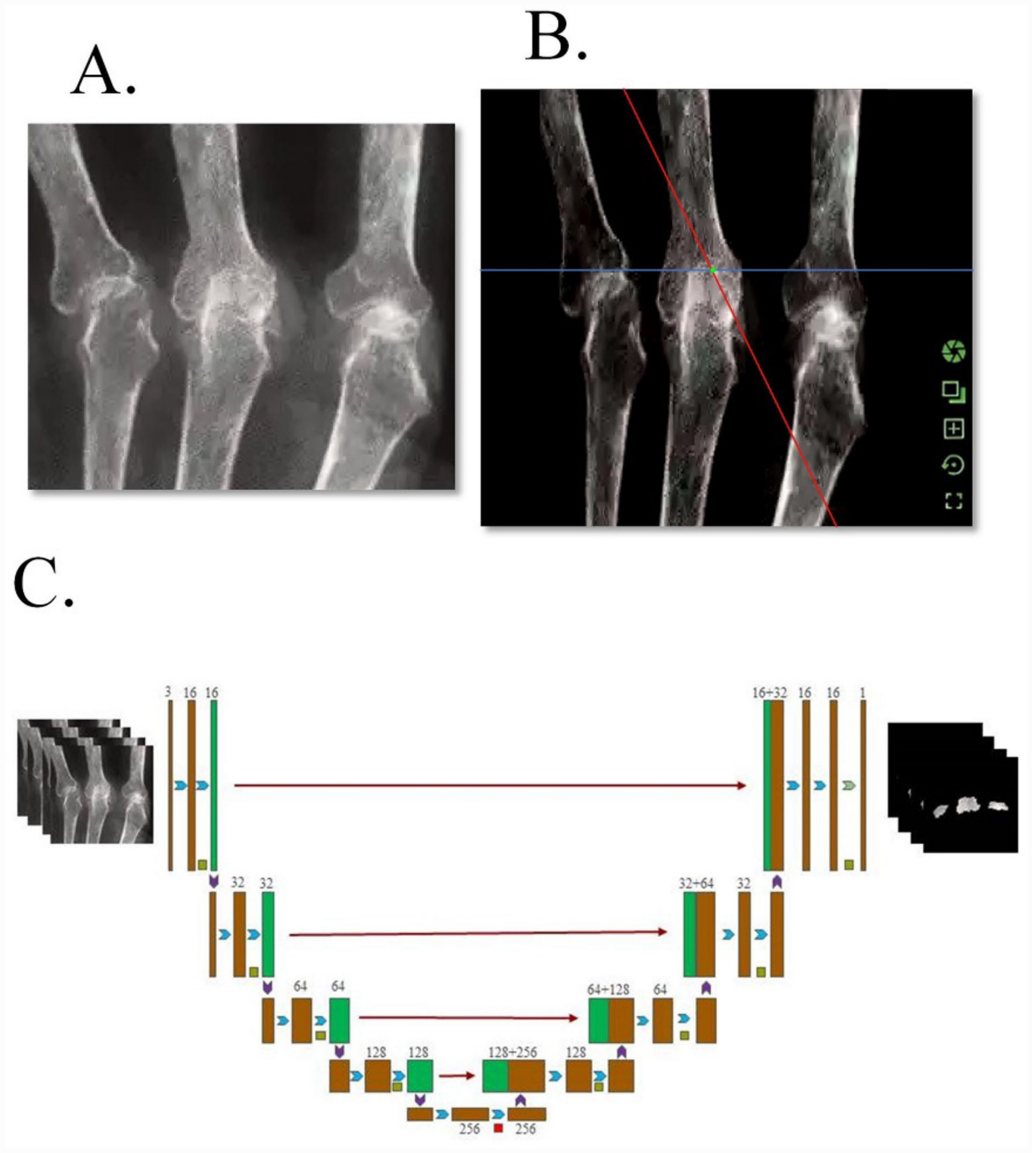
CT segmentation result example. **(A)** Original CT image, **(B)** gold standard segmentation map determined jointly by two senior physicians, **(C)** model-predicted segmentation map.

CT Segmentation Result Example (Figure 6) shows a three-image comparison of the original CT image, the physician’s gold standard segmentation map, and the model’s predicted segmentation map, with key BE regions annotated (indicated by red arrows), visually presenting the model’s segmentation performance.

### 3.6 Validation of consistency between BE severity scoring and clinical assessment

Based on the segmentation results of the model, the overall and local BE severity scores were calculated for 16 RA patients. The overall score ranged from 12.3 to 89.7 points. Among the local scores, the wrist joint had the highest average score, while the PIPJ had the lowest (Table 10). According to the overall scoring criteria, among the 16 patients, there were 0 cases in the no BE cohort, 4 cases (25.0%) in the mild BE cohort, 7 cases (43.75%) in the moderate BE cohort, and 5 cases (31.25%) in the severe BE cohort. In combination with follow-up data, 3 cases (18.75%) were identified in the rapid progression cohort, and 2 cases (12.5%, both with severe wrist joint erosion) were identified in the special site erosion cohort.

**Table 10:** Results of BE severity scoring in RA patients.

Score type	Score range (points)	Mean (points)	Median (points)
Overall severity score	12.3–89.7	56.4 ± 21.8	58.2
Local severity score-wrist joint	15.7–92.4	62.3 ± 23.1	64.5
Local severity score-MPJ	10.5–85.6	53.7 ± 20.9	55.1
Local severity score-PIPJ	8.2–78.9	47.6 ± 19.5	49.3

According to the overall scoring criteria, among the 16 patients, there were 0 in the no BE group, 4 (25.0%) in the mild BE group, 7 (43.75%) in the moderate BE group, and 5 (31.25%) in the severe BE group. Combined with follow-up data (≥3 CT follow-ups, interval ≥6 months), 3 rapid progression cases were identified (18.75%, annual score growth rate ≥30%), and 2 special site erosion cases (12.5%, both with severe wrist erosion, erosion volume ≥40% of wrist bone tissue volume).

Clinical consistency analysis showed that the ICC between model estimation results and physician gold standard annotations was 0.85 (95% CI: 0.79–0.90), indicating high consistency. Bootstrap sampling (sampling times = 1,000) was used to verify the stability of correlation results, showing the PCC fluctuation range between the model’s overall severity score and DAS28 score was 0.72–0.83, and the PCC fluctuation range between wrist joint local score and DAS28 was 0.75–0.86, indicating the results had certain stability, but due to sample size limitations, further validation with larger samples is still needed.

The PCC between the model’s overall severity score and DAS28 score was 0.78 (95% CI: 0.61–0.88, *p* < 0.001). Among local scores, wrist joint score showed the highest correlation with DAS28 (*r* = 0.81, 95% CI: 0.65–0.90, *p* < 0.001). The PCC between MPJ and PIPJ scores and DAS28 were 0.75 (95% CI: 0.57–0.86, *p* < 0.001) and 0.71 (95% CI: 0.52–0.84, *p* = 0.002), respectively (Table 11).

**Table 11:** Validation of clinical consistency of model prediction results.

Validation indicator	Value	95% CI	*P*
ICC value (model-gold standard annotation)	0.85	0.79–0.90	—
PCC (overall score-DAS28)	0.78	0.61–0.88	<0.001
PCC (wrist joint local score-DAS28)	0.81	0.65–0.90	<0.001
PCC (MPJ local score-DAS28)	0.75	0.57–0.86	<0.001
PCC (PIPJ local score-DAS28)	0.71	0.52–0.84	0.002

## 4 Discussion

This study first constructed an RA-related inflammation core gene set containing 826 genes. Differential expression analysis screened 128 DEGs. Intersection with the inflammation core gene set yielded 67 RA-related irDEGs, with 52 upregulated and 15 downregulated, consistent with the pathological nature of RA as an autoimmune inflammatory disease (15). Further prediction revealed that among the 67 irDEGs, 42 contained potential rG4s structures, with a positive rate of 62.69%. The rG4s positive rate in downregulated irDEGs (73.33%) was higher than in upregulated genes (59.62%). These rG4s structures were mainly distributed in the mRNA 3’UTR (45.31%) and CDS regions (36.72%), with the core structures predominantly consisting of 3 G-quadruplexes and loop lengths of 3–5 nt. Functional enrichment analysis showed that irDEGs containing rG4s structures were significantly enriched in 19 core pathways, with the TNF pathway as the network hub, enriched with 13 key genes and the lowest FDR value (1.8 × 10^−12^).

At the imaging analysis level, the optimized U-Net CNN model constructed based on the PyTorch framework achieved precise segmentation of BE regions on the test set, with pixel-level accuracy of 98.7% ± 0.6%, Dice Similarity Coefficient (DSC) of 0.863 ± 0.042, and stable segmentation performance in the wrist, MPJ, and PIPJ. The BE severity scoring system based on model segmentation results showed that its overall score had a PCC of 0.78 with DAS28 (*p* < 0.001). Among local scores, wrist joint score showed the highest correlation with DAS28 (*r* = 0.81, *p* < 0.001). The ICC between model predictions and physician gold standard annotations was 0.85, indicating the model’s quantitative results possessed both accuracy and clinical relevance.

Based on these results, this study proposes the following biological hypothesis: rG4s structures may be involved in the pathological process of RA bone erosion through post-transcriptional regulation. From the gene distribution characteristics, the higher positive rate of rG4s in downregulated irDEGs suggests they may maintain the low expression levels of these genes by stabilizing mRNA secondary structure or inhibiting translation initiation (16). For upregulated irDEGs such as TNF and IL6, rG4s may promote their expression by participating in post-transcriptional modification or binding regulatory proteins (17). This differential distribution suggests rG4s may have bidirectional regulatory effects on gene expression, but specific regulatory modes require experimental confirmation.

From the structural characteristics of rG4s, their high enrichment in the mRNA 3’UTR and CDS regions aligns with their potential functional localization: rG4s in the 3’UTR may be involved in mRNA stability regulation or compete with microRNAs for binding sites (18), while rG4s in the CDS may affect ribosome binding efficiency or peptide synthesis. Furthermore, the predominance of core structures with 3 G-quadruplexes and 3–5 nt loop lengths suggests this configuration may be the optimal conformation for maintaining their stability and functional activity, providing structural reference for future exploration of rG4s-targeted intervention.

Functional enrichment analysis showed that irDEGs containing rG4s structures were concentratedly enriched in core pathways such as TNF, IL-17, and osteoclast differentiation, with the TNF pathway as the network hub, directly associated with 7 pathways. It is speculated that rG4s may mediate the cascade of inflammatory pathways by regulating the expression of core genes such as TNF, IL6, and MMP9: The TNF gene contains 2 rG4s structures, and its high expression can directly activate osteoclasts and release downstream factors like IL-17 and MMP9; the MMP9 gene contains 5 rG4s structures, and its encoded matrix metalloproteinase can degrade bone matrix, accelerating the bone erosion process (19). This hypothesis links rG4s structures to core pathological pathways of RA bone erosion. However, the direct regulatory effects of rG4s on these genes still require future validation through *in vitro* experiments such as rG4s stabilizer/disruptor intervention experiments and luciferase reporter gene assays (20).

The core contribution of this study lies in the parallel construction of RA bone erosion imaging quantification and a molecular candidate gene set. At the imaging level, the optimized U-Net CNN model addressed the issues of subjectivity and inefficiency in traditional CT assessment. Its segmentation performance remained stable across different joint sites, especially with a wrist DSC of 0.881, providing an efficient and precise quantitative tool for BE in clinical practice. The significant correlation between model scores and DAS28 indicates imaging quantification results can indirectly reflect patients’ inflammatory activity levels, providing objective reference for disease staging and treatment adjustment. At the molecular level, this study screened 42 RA-related irDEGs containing rG4s structures, clarified their rG4s structural features and functional enrichment pathways, providing a candidate gene set for future exploration of post-transcriptional regulatory mechanisms in bone erosion. Previous research on rG4s in RA bone erosion was relatively scarce. This study is the first to reveal the association between rG4s and RA inflammation core genes, providing a new research direction for understanding bone destruction mechanisms from the post-transcriptional regulatory level.

This study has the following limitations: First, the sample size was limited and it was a single-center retrospective study (148 RA cases, 49 healthy controls). Model validation relied solely on internal data, lacking external independent cohort validation, which may lead to overfitting risks and requires improvement in result generalizability. Second, the functional roles of rG4s were based solely on bioinformatics predictions and not confirmed by *in vitro* experiments; related regulatory mechanisms remain hypothetical. Third, the deep learning model was not compared with classical or other CNN methods. The model’s innovation mainly lies in structural optimization rather than entirely novel architecture design. Subsequent methodological comparisons are needed to clarify the model’s advantages. Fourth, the weight allocation for the BE severity score, although referencing literature and expert opinions, was not validated with large-sample data; the rationality of weights still requires further optimization.

Addressing these limitations, future research could expand in the following directions: Increase sample size and incorporate multi-center data for external validation to enhance model generalization; validate the regulatory effects of rG4s on core genes like TNF and IL6 through experiments such as rG4s stabilizer/disruptor intervention and RNA immunoprecipitation; compare the model with other segmentation models (e.g., U-Net++, SegNet) to optimize model structure for improved small lesion recognition; re-optimize BE score weight allocation using machine learning algorithms based on large-sample data to enhance the clinical applicability of the scoring system. Additionally, future exploration could combine rG4s-targeted intervention with imaging monitoring to provide new strategies for precise treatment of RA bone erosion.

## 5 Conclusion

This study, through deep learning analysis of CT images, constructed an optimized U-Net CNN model capable of precise quantification of RA bone erosion. Its quantitative results were highly correlated with clinical disease activity, providing an efficient tool for clinical assessment. Simultaneously, using bioinformatics methods, it screened RA-related irDEGs containing rG4s structures, clarifying their structural features and functional enrichment pathways, providing hypotheses and candidate targets for exploring post-transcriptional regulatory mechanisms of bone erosion. The results indicate a potential association between CT imaging characteristics of RA bone erosion and the expression of rG4s-regulated inflammation genes, but the direct molecular mechanisms of this association require experimental validation. This study provides important support for precise assessment and mechanism research of RA bone erosion. Subsequent steps are needed to improve research conclusions by expanding sample size and conducting experimental validation.

## Data Availability

The original contributions presented in the study are publicly available. This data can be found here: https://www.ncbi.nlm.nih.gov/#!/home/contact accession number PX937216.
